# Comparative Gene Expression Analysis of Two Mouse Models of Autism: Transcriptome Profiling of the BTBR and *En2*^−/−^ Hippocampus

**DOI:** 10.3389/fnins.2016.00396

**Published:** 2016-08-25

**Authors:** Giovanni Provenzano, Zelia Corradi, Katia Monsorno, Tarcisio Fedrizzi, Laura Ricceri, Maria L. Scattoni, Yuri Bozzi

**Affiliations:** ^1^Laboratory of Molecular Neuropathology, Centre for Integrative Biology, University of TrentoTrento, Italy; ^2^Bioinformatics Core Facility, Centre for Integrative Biology, University of TrentoTrento, Italy; ^3^Neurotoxicology and Neuroendocrinology Section, Department of Cell Biology and Neuroscience, Istituto Superiore di SanitàRome, Italy; ^4^National Research Council Neuroscience InstitutePisa, Italy

**Keywords:** autism, hippocampus, gene expression, microarray, WGCNA, BTBR, Engrailed, mouse

## Abstract

Autism spectrum disorders (ASD) are characterized by a high degree of genetic heterogeneity. Genomic studies identified common pathological processes underlying the heterogeneous clinical manifestations of ASD, and transcriptome analyses revealed that gene networks involved in synapse development, neuronal activity, and immune function are deregulated in ASD. Mouse models provide unique tools to investigate the neurobiological basis of ASD; however, a comprehensive approach to identify transcriptional abnormalities in different ASD models has never been performed. Here we used two well-recognized ASD mouse models, BTBR T^+^ *Itpr3*^*tf*^/J (BTBR) and Engrailed-2 knockout (*En2*^−/−^), to identify conserved ASD-related molecular signatures. *En2*^−/−^ mice bear a mutation within the EN2 transcription factor homeobox, while BTBR is an inbred strain with unknown genetic defects. Hippocampal RNA samples from BTBR, *En2*^−/−^ and respective control (C57Bl/6J and *En2*^+/+^) adult mice were assessed for differential gene expression using microarrays. A total of 153 genes were similarly deregulated in the BTBR and *En2*^−/−^ hippocampus. Mouse phenotype and gene ontology enrichment analyses were performed on BTBR and *En2*^−/−^ hippocampal differentially expressed genes (DEGs). Pathways represented in both BTBR and *En2*^−/−^ hippocampal DEGs included abnormal behavioral response and chemokine/MAP kinase signaling. Genes involved in abnormal function of the immune system and abnormal synaptic transmission/seizures were significantly represented among BTBR and *En2*^−/−^ DEGs, respectively. Interestingly, both BTBR and *En2*^−/−^ hippocampal DEGs showed a significant enrichment of ASD and schizophrenia (SCZ)-associated genes. Specific gene sets were enriched in the two models: microglial genes were significantly enriched among BTBR DEGs, whereas GABAergic/glutamatergic postsynaptic genes, FMRP-interacting genes and epilepsy-related genes were significantly enriched among *En2*^−/−^ DEGs. Weighted correlation network analysis (WGCNA) performed on BTBR and *En2*^−/−^ hippocampal transcriptomes together identified six modules significantly enriched in ASD-related genes. Each of these modules showed a specific enrichment profile in neuronal and glial genes, as well as in genes associated to ASD comorbidities such as epilepsy and SCZ. Our data reveal significant transcriptional similarities and differences between the BTBR and *En2*^−/−^ hippocampus, indicating that transcriptome analysis of ASD mouse models may contribute to identify novel molecular targets for pharmacological studies.

## Introduction

Autism spectrum disorders (ASD) are a family of neurodevelopmental disorders characterized by a high degree of genetic heterogeneity. Recent advances in genetics and genomics allowed to attribute the heterogeneous clinical manifestations of ASD to shared pathophysiological processes (de la Torre-Ubieta et al., 2016). Integrative analysis of large-scale genetic data revealed distinct gene networks affected in ASD, mainly related to the formation and function of brain synapses. Network-based analysis of large-scale transcriptome data also highlighted that co-expression modules related to synapse development, neuronal activity and immune function are deregulated in ASD (Voineagu et al., 2011; Gupta et al., 2014; de la Torre-Ubieta et al., 2016). Thus, pathophysiological processes in ASD seem to converge on specific molecular pathways and networks, with a clear interplay between immune and synaptic functions (Estes and McAllister, 2015).

Gene transcriptional profiling in ASD is mainly performed on post-mortem brain samples, but the restricted availability of human ASD brain tissues represents a significant challenge. For this reason, ASD mouse models provide a unique tool to identify conserved pathological mechanisms at the gene expression level. BTBR T^+^ *Itpr3*^*tf*^/J (BTBR) is an inbred strain of mice that incorporates behavioral phenotypes relevant to all diagnostic symptoms of ASD, including reduced social interactions in juveniles and adults, repetitive self-grooming and an unusual pattern of ultrasonic vocalizations resembling the atypical vocalizations seen in some autistic children (Scattoni et al., 2008, 2013). Moreover, BTBR mice show a severely reduced hippocampal commissure and absent corpus callosum (Wahlsten et al., 2003). Noteworthy, corpus callosum abnormalities have been reported in autistic individuals (Egaas et al., 1995; Alexander et al., 2007). However, it is important to emphasize that genetic abnormalities causing behavioral deficits in BTBR mice are still under investigation (Jones-Davis et al., 2013).

Engrailed-2 (EN2) is a homeodomain transcription factor involved in regionalization and patterning of the midbrain and hindbrain regions (Joyner et al., 1991). Genome-wide association studies revealed that EN2 is a candidate gene for ASD (Benayed et al., 2009), and an abnormal expression and methylation profile of the EN2 gene has been reported in the cerebellum of ASD patients (James et al., 2013, 2014; Choi et al., 2014). Mice lacking the homeobox domain of *En2* (*En2*^*hd*/*hd*^ mice; Joyner et al., 1991; here referred to as *En2*^−/−^) display neuropathological changes related to ASD. These defects include cerebellar hypoplasia and reduced number of Purkinje neurons (Joyner et al., 1991; Kuemerle et al., 1997), defective GABAergic innervation in the forebrain (Sgadò et al., 2013a; Allegra et al., 2014; Provenzano et al., 2014), reduced monoaminergic innervation to the forebrain (Brielmaier et al., 2014; Genestine et al., 2015; Viaggi et al., 2015) and ASD-like behavioral traits such as decreased sociability, spatial learning deficits, and increased seizure susceptibility (Cheh et al., 2006; Tripathi et al., 2009; Brielmaier et al., 2012; Provenzano et al., 2014).

So far, a comprehensive approach to identify common and distinct abnormalities in ASD models has been conducted by brain imaging-based neuroanatomical phenotyping (Ellegood et al., 2015; Ellegood and Crawley, 2015). However, few efforts have been made to identify conserved genes signatures at the transcriptome level in brain tissues from ASD mice. The hippocampal and cortical transcriptome has been examined in BTBR mice (Daimon et al., 2015; Kratsman et al., 2016), while we evaluated the gene expression signature of the *En2*^−/−^ hippocampus and cerebellum (Sgadò et al., 2013b). Similar studies have been performed on brains from *Fmr1* (Prilutsky et al., 2015) and *Pten* (Tilot et al., 2016) mutant mice. However, a comprehensive approach to identify transcriptional abnormalities in ASD mouse models has never been performed so far. Here we compared the transcriptome profile of the BTBR and *En2*^−/−^ hippocampus, and describe common and distinct transcriptional signatures for these two ASD mouse models.

## Materials and methods

### Animals

Experiments were conducted in conformity with the European Community Directive 2010/63/EU and were approved by the Animal Welfare Committee of University of Trento, Istituto Superiore di Sanità and Italian Ministry of Health. Animals were housed in a 12 h light/dark cycle with food and water available *ad libitum*, and all efforts were made to minimize animal suffering during the experiments. *En2* mutants were originally generated on a mixed 129Sv x C57BL/6 genetic background (Joyner et al., 1991) and then backcrossed at least five times into a C57BL/6 background. *En2*^+/+^ and *En2*^−/−^ mice used in this study were obtained by heterozygous mating (*En2*^+/−^ x *En2*^+/−^) and genotyped by PCR as previously described (Sgadò et al., 2013a). BTBR T^+^ *Itpr3*^*tf*^/J (BTBR) and C57Bl/6J (B6) inbred mice were purchased from the Jackson Laboratory (Bar Harbour, ME, USA) and bred in the mouse vivarium of the Istituto Superiore di Sanità (Rome, Italy). A total of 32 adult (3–5 months old) mice were used: 4 BTBR and 4 B6 mice for microarray experiments, and 6 mice per strain/genotype (BTBR, B6, *En2*^+/+^ and *En2*^−/−^*)* for quantitative RT-PCR.

### Microarrays and single-gene differential expression analysis

In this study, we aimed to identify common and distinct molecular signatures across the hippocampi of BTBR and *En2*^−/−^ mice. To obtain a comparable and reliable hippocampal transcriptomic profile of BTBR mice and reduce any source of variability during microarray analysis, which might arise from pre-scanning and/or post-scanning steps (Kadanga et al., 2008), we applied the same experimental procedures previously described for the transcriptome analysis of *En2*^−/−^ hippocampus (Sgadò et al., 2013b). Briefly, hippocampal RNAs from BTBR and B6 mice (*n* = 4 per experimental group) were purified using standard column purification according to the manufacturer's protocol (RNAeasy Mini Kit, QIAGEN). RNA quality was analyzed by microfluidic gel electrophoresis on RNA 6000 NanoChips using the Agilent 2100 Bioanalyzer. Only RNA with a high (>9) RNA integrity number was selected and used for subsequent retrotranscription, labeling, and array hybridization according to Agilent protocols. Mouse gene expression arrays (Agilent 4X44K slides) were hybridized and scanned with the Agilent microarray station. The images obtained from the microarray scanner were analyzed with Agilent Feature Extraction version 10.7.3.1. Hippocampal gene expression dataset of BTBR and B6 mice have been deposited in the NCBI's Gene Expression Omnibus (GEO) database (accession number GSE81501). Intensity values were processed with Agi4x44PreProcess using default parameters to remove low-quality probes. Signals were then normalized by means of the quantile normalization method. Multiple replicas of the same probes were summarized using the median. To evaluate differential expression, the Rank Product (RP) non-parametric method was used (Sgadò et al., 2013b). The RP is equivalent to calculating the geometric mean rank with a statistical method (average rank) that is slightly more sensitive to outlier data and puts a higher premium on consistency between the ranks in various lists.

### Quantitative RT-PCR (qRT-PCR)

Total RNAs were extracted by Trizol reagent (Invitrogen) from explanted hippocampi. DNase-treated RNAs were purified by RNA extraction RNAeasy Kit (QIAGEN). cDNA was synthesized from pooled RNAs by SuperScript VILO cDNA Synthesis Kit (Invitrogen) according to the manufacturer' instructions. qRT-PCR was performed in a C1000 Thermal Cycler (Bio-Rad) with real-time detection of fluorescence, using the KAPA SYBR FAST Master Mix reagent (KAPA Biosystems). Mouse mitochondrial ribosomal protein L41 (mRPL41) was used as a standard for quantification. Primer sequences (Eurofins Genomics) are reported in Supplementary Table 1. Ratios of comparative concentrations of each mRNA with respect to L41 mRNA were then calculated and plotted as the average of three independent reactions (technical replicates) obtained from each RNA. Expression analyses were performed using the CFX3 Manager (Bio-Rad) software (Sgadò et al., 2013b). Statistical analysis of qRT-PCR was performed with Prism 6 (GraphPad) software. Values were expressed as mean ± s.e.m and quantitative gene expression differences between each autistic mouse (BTBR and *En2*^−/−^) strain and their respective controls (B6 and *En2*^+/+^) were assessed by Student's *t*-test, with the level of statistical significance set at *p* < 0.05.

### Phenotype/pathway ontology and enrichment analysis on DEGs

DEGs in BTBR and *En2*^−/−^ hippocampi were analyzed for “phenotype ontology” using Enrichr (http://amp.pharm.mssm.edu/Enrichr/). Enrichr is an enrichment analysis web-based tool providing various types of gene-set libraries, including the knockout mouse phenotypes ontology developed by the Jackson Lab from their Mouse Genome Informatics-Mammalian Phenotype (MGI-MP) browser (Blake et al., 2009). Mammalian phenotypes predefined by MGI are sorted by *z*-score considering only terms with an adjusted *p*-value less than 0.05. Phenotype/pathway ontology of *En2*^−/−^ hippocampal DEGs was performed on our previous dataset from *En2*^+/+^ and *En2*^−/−^ adult mice, already deposited in NCBI's GEO database (accession number GSE51612; Sgadò et al., 2013b). In order to visualize enriched Kyoto Encyclopedia of Genes and Genomes (KEGG) pathways, we used DAVID v6.7 (http://david.abcc.ncifcrf.gov/). To focus the functional analysis on hippocampal expressed genes we used as background a list of tissue-specific expressed genes for both autistic (BTBR and *En2*^−/−^) strains. These background lists were obtained by filtering the genes by the normalized expression values and excluding the ones with the lowest expression levels (< 10th percentile).

We also performed the direct enrichment analysis of different gene set categories present in the DEGs from BTBR and *En2*^−/−^ mice, using the hypergeometric test present in R (*P*-value cut-off 0.05). The background used to compute the enrichments was the same used for KEGG pathway analysis (see below for the gene lists used for enrichment analyses).

### Weighted correlation network analysis (WGCNA) and enrichment on co-expression modules

Microarray data from the BTBR and *En2*^−/−^ hippocampi were analyzed with the WGCNA R package in order to find highly correlated modules of co-expressed genes (Langfelder and Horvath, 2008). Two different batches of samples, autistic (BTBR and *En2*^−/−^) vs. control (B6 and *En2*^+/+^), were used to obtain an appropriate number for the WGCNA analysis. All samples were normalized and filtered using the same method described above. The R program ComBat (Johnson et al., 2007) included in the SVA package (Leek et al., 2012) was used to remove the batch effect present on different chips, in which samples were run. In order to keep the most informative probes, only the top 20% (in terms of per-probe variance) were used as input for the WGCNA analysis. The WGCNA method is based on soft threshold approach in order to obtain an adjacency matrix that describes the relations among the genes (Zhang and Horvath, 2005). The scale-free criterion is used to select the power to apply to the correlations in order to obtain the adjacency matrix, a power of 11 was chosen in this study. The Topological Overlap Measure (TOM) is then calculated starting from the previously obtained adjacency matrix. TOM is a highly robust measure of network interconnectedness (proximity) and expresses the strength characterizing the connection between each pair of genes. Genes with high TOM are clustered into co-expression modules. kME (representing the connection strength of each gene in each module) is then calculated as the correlation of each gene to the module eigengene (ME, defined as the first principal component of the expressions of the genes within the module). The ME defines measures of module membership (MM), which quantify how close a gene is to a given module eigengene. For each expression profile, Gene Significance (GS) was calculated as the absolute value of the correlation between expression profile and trait (autistic phenotype). The statistical significance of MM and GS (denoted as p.MM and p.GS) is carried out from the correlation test *p*-value of the WGCNA package. All values are tabulated in Supplementary Table 3. Given autism is a complex and very heterogeneous group of disorders, simple correlation of the modules with the trait produced meaningless results. A different approach was hence used: the modules were ranked based on their enrichment in term of genes present in the SFARI list. Then, modules significantly enriched in ASD-associated genes were functionally characterized for enrichment in markers of specific synapse, cell and disease types, using the same method described above. Moreover, to evaluate the biological and functional relevance of ASD-associated modules we used the Enrichr web tool. We analyzed the overrepresentation of the biological processes GO category for each gene list of modules.

### Gene lists used for enrichment analyses

SFARI database (https://gene.sfari.org/autdb/Welcome.do) was used to calculate enrichment with ASD-associated genes on BTBR and *En2*^−/−^ hippocampal DEGs. For disease annotations, we used the following gene sets from Autworks database (http://autworks.hms.harvard.edu): epilepsy, parkinsonian disorders and schizophrenia. For searching the recurrence and overlaps with FMRP-associated genes, we used a set of 842 genes (herein termed “FMRP Interacting Genes”), from a crosslinking and immunoprecipitation (CLIP) experiment (Darnell et al., 2011). Other gene lists used in this study (neuronal markers, astrocyte markers, type 1 microglial markers, type 2 microglial markers, oligodendrocyte markers, postsynaptic density, asdM12, asdM16) are available at http://www.arkinglab.org/resources. GABAergic synapse, glutamatergic synapse, dopaminergic synapse lists were taken from http://www.genome.jp/kegg/. In addition, the following gene lists were compiled from MsigDB (http://www.broadinstitute.org/gsea/msigdb/collections.jsp#C1): GABAergic presynaptic markers (GABA synthesis, release, reuptake and degradation); GABAergic postsynaptic markers (GABA A receptor activation, GABA B receptor activation, GABA receptor activation); glutamaergic presynaptic markers (glutamate neurotransmitter release cycle); glutamaergic postsynaptic markers (glutamate receptor activity; glutamate signaling pathway); dopaminergic markers (dopamine neurotransmitter release cycle).

## Results

**Figure 1:**
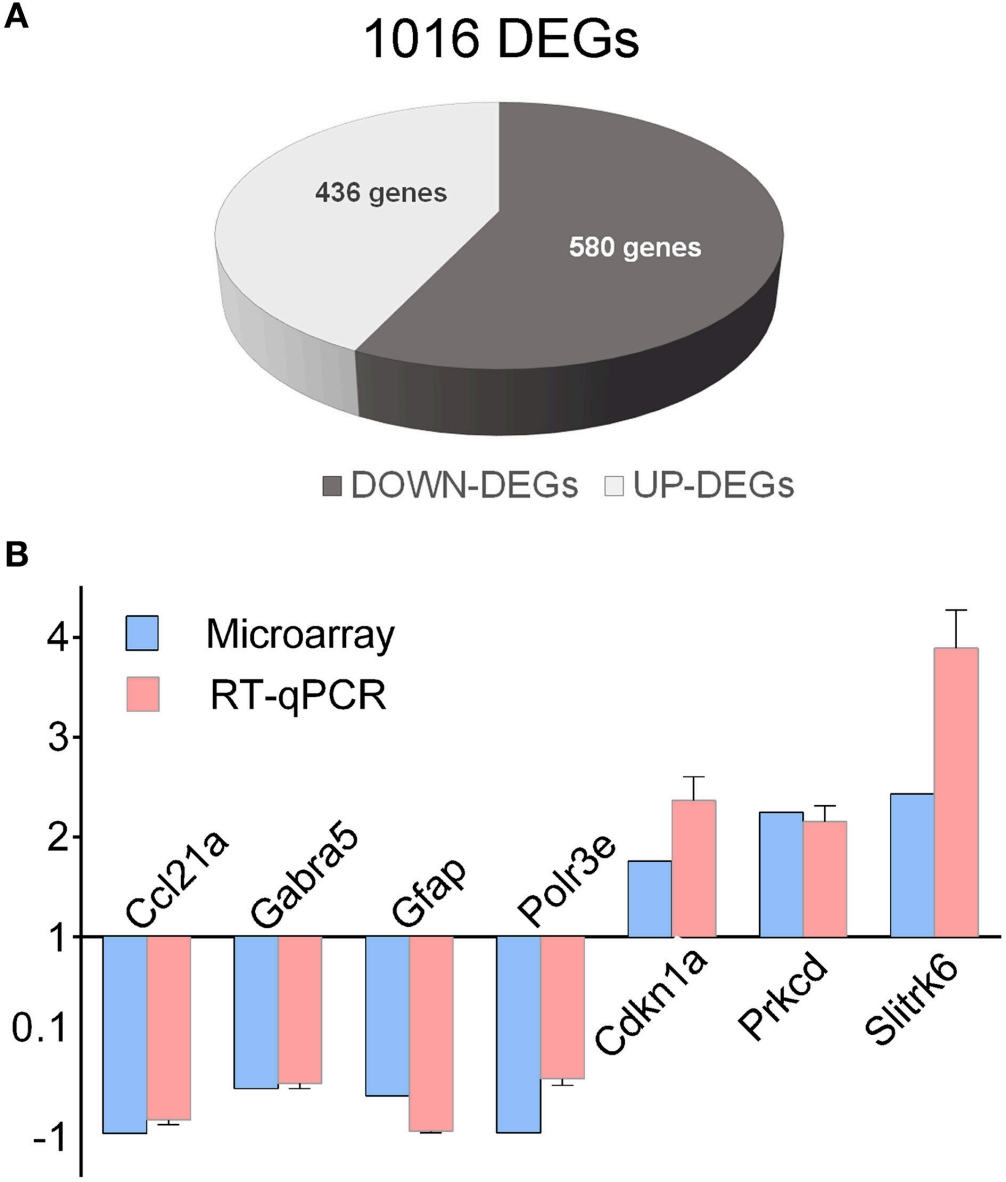
**Differentially expressed genes in the BTBR hippocampus. (A)** Schematic representation of BTBR hippocampal DEGs, as compared to control B6 mice. A total of 1016 DEGs were identified, (580 down-regulated, 436 up-regulated). **(B)** qRT-PCR validation of differentially expressed genes. qRT-PCR results for all the evaluated genes were in agreement with microarray results. Values are expressed as each gene/L41 comparative quantitation ratios normalized on the expression of WT (mean ± s.e.m of three replicates from pools of six animals per genotype; *p* < 0.05, Student's *t*-test, BTBR vs. B6).

Recently, we showed by transcriptome analysis that several genes related to ASD, abnormal synaptic transmission and GABA signaling are markedly deregulated in the hippocampus of *En2*^−/−^ mice (Sgadò et al., 2013b). Here we extended this analysis to the hippocampus of BTBR mice, to test the hypothesis that common and distinct downstream mechanisms may be altered in these two ASD mouse models. Hippocampi from BTBR and B6 control adult mice were assessed for differential gene expression by microarray and bioinformatic analysis, as previously reported for *En2*^−/−^ mice (Sgadò et al., 2013b). We found 1016 differentially expressed genes in the hippocampus of BTBR mice compared to their B6 controls (Figure 1A). Among these, 436 and 580 were up- and down-regulated, respectively. Supplementary Table 2 shows the entire list of genes differentially expressed in the BTBR hippocampus, with fold change, percentage of false prediction (pfp) and *P*-values calculated by RankProd. We next validated microarray findings by qRT-PCR analysis, selecting seven representative genes from the DEGs list. Except for Gabra5, for which decreased protein and mRNA levels were found in the brains of ASD patients (Fatemi et al., 2010), many of the selected genes belong to immune/inflammatory categories. All genes showed statistically significant differential expression in the BTBR hippocampus, as compared to B6 controls [chemokine (C-C motif) ligand 21A (Ccl21a) *p* = 0.0424; gamma-aminobutyric acid (GABA) A receptor, subunit alpha 5 (Gabra5) *p* = 0.0069; glial fibrillary acidic protein (Gfap) *p* = 0.009; polymerase (RNA) E (DNA directed) polypeptide E (Polr3e) *p* = 0.025; cyclin-dependent kinase inhibitor 1A (P21) (Cdkn1a) *p* = 0.0052; protein kinase C, delta polypeptide E (Polr3e) *p* = 0.0029; SLIT and NTRK-like family, member 6 (Slitrk6) *p* = 0.0018] (Figure 1B). In all tested genes the expression difference reported by qRT-PCR significantly correlated with microarray data (Pearson *r* = 0.97, *p* < 0.0003).

**Figure 2:**
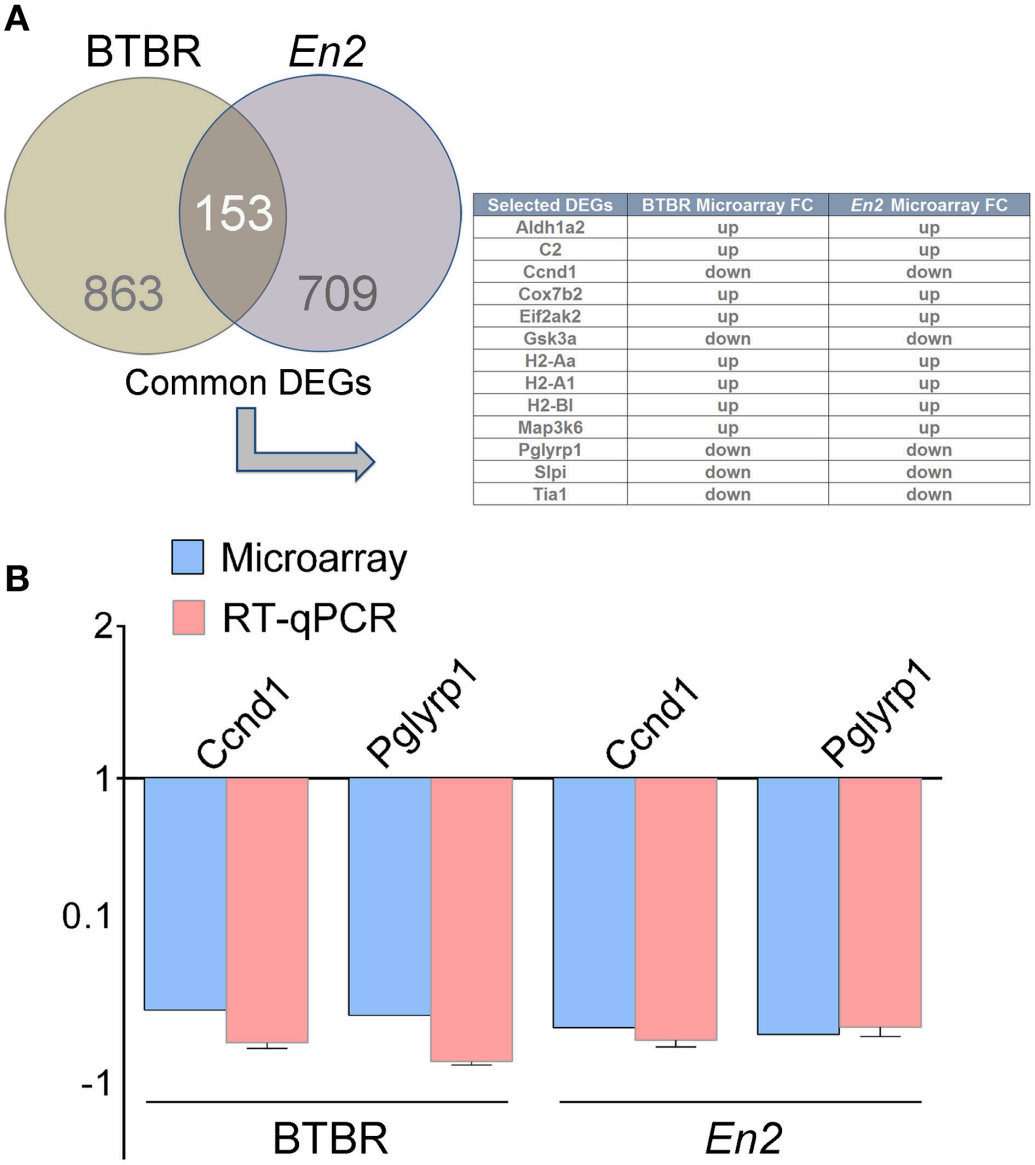
**DEGs commonly expressed in the BTBR and ***En2***^−/−^ hippocampus. (A)** Venn diagram of differentially expressed genes in the BTBR and *En2*^−/−^ adult hippocampus. A total of 1016 and 862 differentially expressed genes were identified in BTBR (brown) and *En2*^−/−^ (gray) mice, respectively. Among these, 153 show differential expression in both strains. The table shows a subset of these common DEGs, all belonging to inflammatory pathways. **(B)** qRT-PCR validation of Ccnd1 and Pglyrp1, two genes differentially expressed in both BTBR and *En2*^−/−^ hippocampi. qRT-PCR results for both genes were in agreement with microarray results. Values are expressed as each gene/L41 comparative quantitation ratios normalized on the expression of respective control (mean ± s.e.m of three replicates from pools of six animals per genotype; *p* < 0.05, Student's *t*-test, BTBR vs. B6 and *En2*^−/−^ vs. *En2*^+/+^).

We next compared the BTBR hippocampal transcriptional profile to that of *En2*^−/−^ mice. We first identified a total number of 153 commonly expressed genes between BTBR and *En2*^−/−^ mice Interestingly, some of the common DEGs are related to inflammatory pathways (Aldh1a2, C2, Ccnd1, Cox7b2, Eif2ak2, Gsk3a, H2-Aa, H2-A1, H2-Bl, Map3k6, Pglyrp1, Slpi, Tia1; Figure 2A). qRT-PCR confirmed the differential expression of two of these genes [cyclin D1 (Ccdn1), *p* = 0.0018 and *p* = 0.0051 for BTBR and *En2*^−/−^ mice, respectively; peptidoglycan recognition protein 1 (Pglyrp1), *p* = 0.0052 and *p* = 0.0284 for BTBR and *En2*^−/−^ mice, respectively] (Figure 2B).

**Figure 3:**
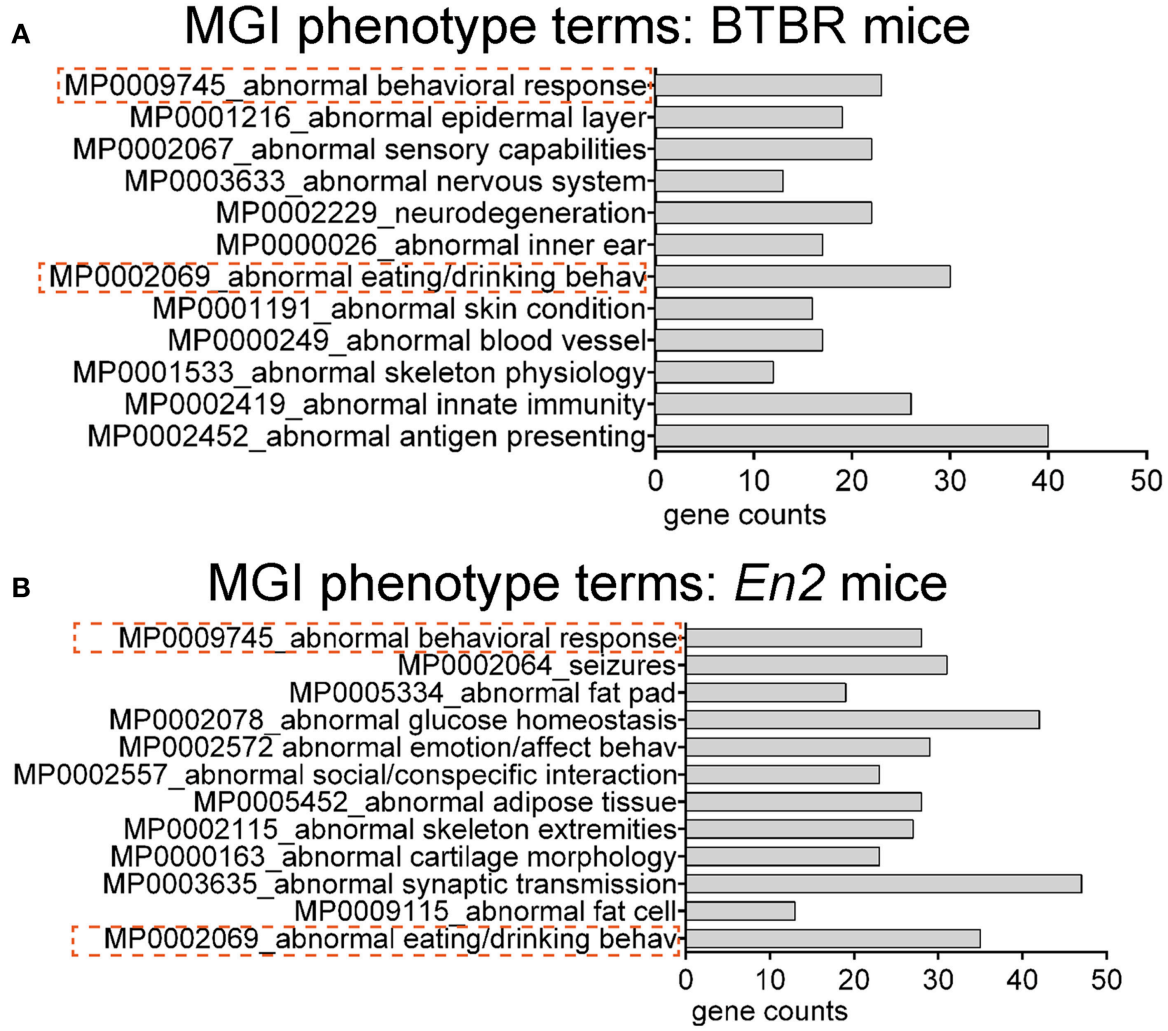
**Overrepresented mouse phenotype categories for differentially expressed genes in BTBR and *En2*^−/−^ hippocampi**. BTBR and *En2*^−/−^ hippocampal DEGs were analyzed for enrichment in phenotype ontology categories using Enrichr, with an adjusted *p* < 0.05. For each category, the number of genes is indicated by the length of the horizontal bars (gene counts). Dashed red lines highlight phenotype categories common to BTBR **(A)** and *En2*^−/−^ **(B)** DEGs.

**Figure 4:**
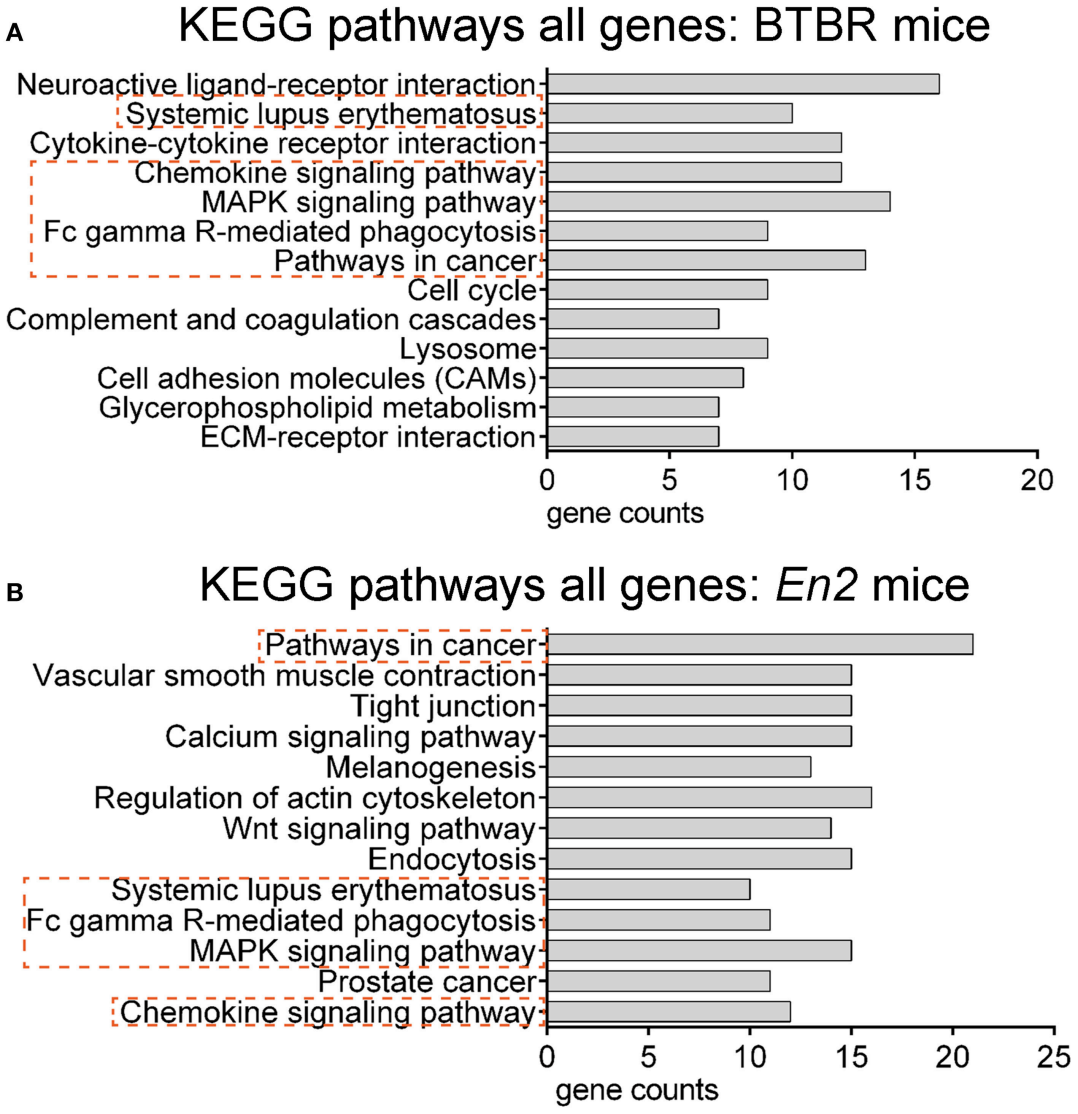
**Overrepresented pathways categories for differentially expressed genes in BTBR and *En2*^−/^^−^ hippocampi**. BTBR and *En2*^−/−^ hippocampal DEGs were analyzed for enrichment in KEGG pathways categories using DAVID, with an adjusted *p* < 0.05. For each category, the number of genes is indicated by the length of horizontal bars (gene counts). Dashed red lines highlight pathways common to BTBR **(A)** and *En2*^−/−^ **(B)** DEGs.

We next explored whether shared functional categories or common pathways were perturbed in both ASD mouse models. To increase accuracy of the functional analysis, we decided to compare the two lists of DEGs using updated databases and different bioinformatic tools, respect to those previously employed for the analysis of the hippocampal trascriptome of *En2*^−/−^ mice (Sgadò et al., 2013b). We tested BTBR and *En2*^−/−^ hippocampal DEGs for enrichment of mouse phenotype terms (Mouse Genome Informatics Mammalian Phenotype Level 4) using Enrichr (Figure 3). Two significantly enriched phenotypes were common to both mouse strains: abnormal behavioral responses and abnormal eating/drinking behavior. Conversely, specific phenotype ontologies enriched in BTBR were abnormal sensory capabilities, neurodegeneration, abnormal innate immunity and abnormal antigen presenting (Figure 3A). For *En2*^−/−^ mice, we confirmed a significant enrichment for terms related to seizure and altered synaptic transmission (Figure 3B), as previously reported (Sgadò et al., 2013b). KEGG pathways analysis for BTBR and *En2*^−/−^ hippocampal DEGs was performed with DAVID, using tissue-specific lists of expressed genes as background for each ASD mouse model (see Materials and Methods). The common pathways that were most significantly enriched across the differentially expressed genes of both ASD mouse models were related to chemokine signaling, MAPK signaling, systemic lupus erythematosus, Fc gamma receptor mediated phagocytosis and pathways in cancer (Figure 4).

**Figure 5:**
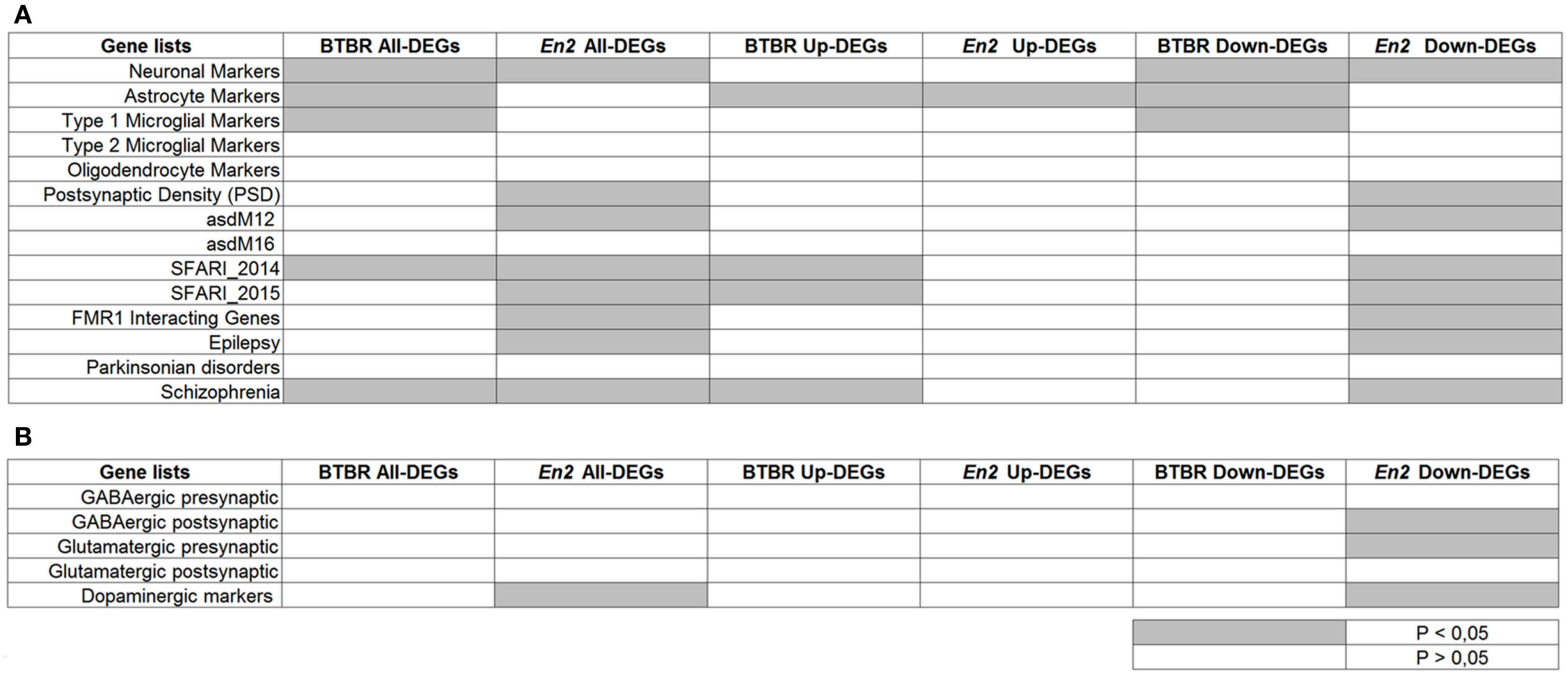
**Enrichment analysis on BTBR and *En2*^−/−^ hippocampal DEGs**. Lists of BTBR and *En2*^−/−^ hippocampal DEGs (all, up-regulated and down-regulated) were analyzed for enrichment in genes belonging to specific categories relevant to the autistic phenotype **(A)** and neuronal subtypes **(B)**. Gene lists were compiled from the literature (see Materials and Methods and references therein). Significance of enrichment is indicated (white, *p* > 0.05; gray, *p* < 0.05).

To further reveal similarities and differences between the BTBR and *En2*^−/−^ hippocampal transcriptome, we tested whether the two lists of DEGs were specifically enriched for cell-type specific markers and genes implicated in neurodevelopmental and neurodegenerative diseases (ASD, SCZ, epilepsy, Parkinson's). Remarkably, when compared to these repositories, BTBR and *En2*^−/−^ hippocampal DEGs were enriched in SCZ- and ASD-related genes according to SFARI 2014 dataset (Figure 5A), consistent with reports showing overlap in candidate genes between ASD and SCZ (Crespi et al., 2010). Furthermore, when we separately analyzed up- and down-regulated genes, we found a significant over-representation of neuronal markers for both BTBR and *En2*^−/−^ down-regulated DEGs; conversely, a significant over-representation of astrocyte markers was found in BTBR and *En2*^−/−^ up-regulated DEGs (Figure 5A). Significant differences were also detected between the two strains. Among the genes down-regulated in the *En2*^−/−^ hippocampus the following terms were significantly enriched: epilepsy, asdM12 (a neuronal module enriched for ASD-associated genes; Voineagu et al., 2011), FMR1 interacting genes, dopaminergic markers, GABAergic postsynaptic markers and glutamatergic presynaptic markers; conversely, type I microglia markers were significantly enriched among BTBR down-regulated genes (Figures 5A,B).

**Figure 6:**
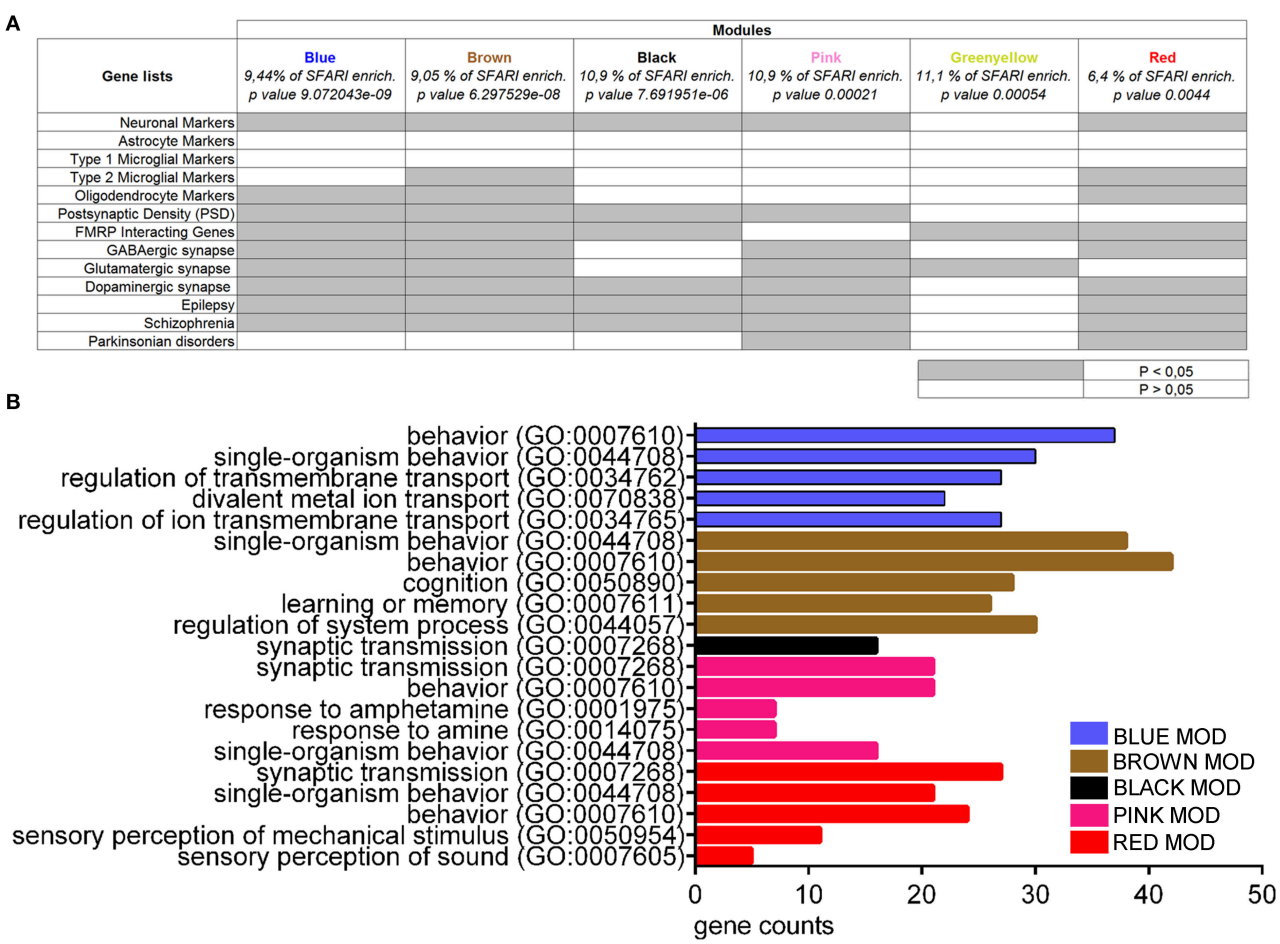
**Enrichment and gene ontology analyses on co-expression modules resulting from WGCNA on BTBR and *En2*^−/−^ hippocampal DEGs**. **(A)** Co-expression modules enriched in SFARI genes were analyzed for enrichment in genes belonging to specific categories relevant to the autistic phenotype, as indicated. Gene lists were as in Figure 5. Significance of enrichment is indicated (white, *p* > 0.05; gray, *p* < 0.05). **(B)** Co-expression modules were analyzed for enrichment in GO categories using Enrichr, with an adjusted *p* < 0.05. For each category, the number of genes is indicated by the length of horizontal bars (gene counts). Modules are indicated in their respective colors. The Greenyellow module is not indicated in the graph since it did not show any significant over-representation of GO terms.

To further investigate the presence of common molecular networks deregulated across autistic mice as compared to their respective controls, we analyzed the entire gene expression dataset (BTBR+*En2*^−/−^ vs. B6+*En2*^+/+^) using WGCNA (Zhang and Horvath, 2005). This analysis allows to identify discrete gene modules based on co-expression profiles (Voineagu et al., 2011). WGCNA identified 18 main modules for groups of genes with high topological overlap, ranging between 28 and 653 probes in size (Supplementary Table 3). The modules were correlated to the disease trait using a linear mixed regression framework and then ranked based on their enrichment in term of genes present in the SFARI list (retrieved on 2015). Six out of the eighteen modules were significantly enriched in ASD-related genes (Blue, *p* = 9.072043e-09; Brown, *p* = 6.297529e-08; Black, *p* = 7.691951e-06; Pink, *p* = 0.00021; Greenyellow, *p* = value 0.00054; Red, *p* = 0.0044) (Figure 6A). We next tested the association of each of the six ASD-enriched modules for enrichment analysis. Except the Greenyellow module, all modules shared a significant enrichment for neuronal markers, as well as for genes associated to epilepsy and SCZ (Figure 6A). The Greenyellow module was enriched only for FMRP-interacting and glutamatergic synapse genes. Brown and Red modules were significantly enriched for oligodendrocyte markers and genes associated with M2-microglial cell states. For a better functional characterization of ASD-associated modules, we also used Enrichr to assess the GO biological process annotation of the six ASD gene-enriched modules. Blue, Black, Pink, and Red modules, which are enriched for neuronal markers (Figure 6A) also contain genes with the GO term “regulation of ion transmembrane transport” or “synaptic transmission” (Figure 6B). The Brown module, one of most enriched for categories (neuronal markers, type 2 microglial markers, oligodendrocyte markers, postsynaptic density, FMRP interacting genes, GABAergic/glutamatergic/dopaminergic synapse, epilepsy, schizophrenia; Figure 6A), showed an over-representation of genes involved in cognition, learning, and memory. Finally, the Greenyellow module did not show any significant over-representation of GO categories (Figure 6B).

## Discussion

### Brief summary of results

In this study, we compared the hippocampal transcriptome of BTBR and *En2*^−/−^ adult mice, two robust animal models of ASD. We identified both common and distinct gene pathways represented in the BTBR and *En2*^−/−^ transcriptome. Common pathways included chemokine and MAP kinase signaling, whereas genes involved in immune dysfunction and abnormal synaptic transmission were specifically represented among BTBR and *En2*^−/−^ DEGs, respectively. Both BTBR and *En2*^−/−^ hippocampal DEGs showed a significant enrichment of ASD and SCZ-associated genes, with specific gene sets enriched in the two models (glial genes in BTBR; GABAergic, glutamatergic FMRP-related and epilepsy-related genes in *En2*^−/−^). Finally, network analysis (WGCNA) performed on BTBR and *En2*^−/−^ hippocampal DEGs together identified six modules significantly enriched in ASD-associated genes, with specific enrichment profile in neuronal, glial, epilepsy-related, and SCZ-associated genes.

### Common pathways deregulated in the BTBR and *En2^−/−^* hippocampus

Our comparative microarray analysis revealed that 155 genes (out of a total of 44,000) are differentially expressed in both BTBR and *En2*^−/−^ hippocampus (Figure 1), indicating that a very low of number of genes (0.35%) are commonly deregulated among these two ASD mouse models. Nevertheless, ontology analyses for phenotypes and cellular pathways revealed that some common gene categories are significantly over-represented among the genes differentially expressed in the BTBR and *En2*^−/−^ hippocampus, as compared to their respective controls (B6, *En2*^+/+^ mice). Mammalian Phenotype ontology revealed that genes involved in abnormal behavioral responses are significantly deregulated in both mouse models (Figure 2). Most importantly, KEGG pathways analysis showed that genes related to immune response and inflammation are significantly deregulated in both BTBR and *En2*^−/−^ hippocampus (KEGG pathways: chemokine signaling, MAPK signaling, systemic lupus erythematosus, Fc gamma receptor mediated phagocytosis; Figure 3). This is in line with our current knowledge about the role of the immune system in ASD pathogenesis. Genetic studies indicate that several genes encoding components of the immune system are associated to ASD. In addition, data from both ASD individuals and animal models indicate a significant dysregulation of immune processes in ASD, such as up-regulation of pro-inflammatory cytokines/chemokines, increased expression of major histocompatibility complex, and activation of microglia in the brain (reviewed in Estes and McAllister, 2015). Significantly increased levels of pro-inflammatory cytokines (IL-1β, IL-6) and microglia activation have been reported in BTBR brains (Heo et al., 2011; Onore et al., 2013; Zhang et al., 2013), similarly to what observed in post-mortem brain tissue from ASD patients (Vargas et al., 2005; Li et al., 2009; Suzuki et al., 2013).

It is important to point out that, although the B6 strain is commonly used to compare with BTBR in molecular and behavioral studies (reviewed in Silverman et al., 2010), it exists the possibility that it is not an ideal or sufficient control for BTBR in gene expression studies. Indeed, linkage disequilibrium studies showed that B6 and BTBR mice are on different branches of the mouse genetic tree (Petkov et al., 2005). Thus, further genetic characterization is needed to identify the ideal control strain for inbred BTBR mice. In the absence of such a control, we conformed to previous studies using B6 as a control for BTBR in transcriptional profiling analyses (Daimon et al., 2015).

Differently from what reported in this study, our previous transcriptome profiling revealed a significant deregulation of immune- and inflammation-related genes in the cerebellum but not hippocampus of *En2*^−/−^ adult mice (Sgadò et al., 2013b). One possible explanation is that our previous gene ontology analysis of the *En2*^−/−^ hippocampal transcriptome was performed using different databases than those used for the present study. Preliminary unpublished data from our laboratory however indicate that the expression of several immune mediators (IL-1β, TNF-α, toll-like receptor 2, CCL2, CCL5) is significantly deregulated in the hippocampus (as well as neocortex and cerebellum) of *En2*^−/−^ adult mice.

Our enrichment analyses revealed that both sets of BTBR and *En2*^−/−^ hippocampal DEGs are enriched in ASD-related genes according to the SFARI 2014 dataset. *En2*^−/−^ hippocampal DEGs also showed a significant enrichment in genes contained in two additional datasets of autism-related genes (asdM12 and SFARI15). This confirms that both BTBR and *En2*^−/−^ mice are valuable mouse models to investigate ASD-relevant gene signatures. Most importantly, these enrichment analyses showed that both BTBR and *En2*^−/−^ DEGs dataset are significantly enriched in SCZ-associated genes (Figure 5). This is in agreement with recent results from large-scale genomic studies showing that ASD and SCZ are neurodevelopmental disorders characterized by overlapping genetics and phenotypes (Murdoch and State, 2013). Genetic lesions at the origin of these disorders are thought to affect brain circuit formation and synaptic function during embryonic and/or postnatal development, ultimately leading to a varying range of (partially overlapping) pathological behaviors in ASD and SCZ. Recent genomic studies show that control subjects carrying copy-number variants (CNVs) conferring risk of ASD or SCZ poorly perform in cognitive tests (Stefansson et al., 2014), suggesting that conserved genetic mechanisms might underlie shared co-morbidities in these two neurodevelopmental disorders.

Enrichment analyses also showed that both sets of BTBR and *En2*^−/−^ hippocampal DEGs are enriched in neuronal and astrocyte markers. The importance of glial cells in ASD pathophysiology was initially suggested by RNA sequencing studies showing a significant enrichment of glial genes belonging to immune/inflammatory categories in ASD brain tissues (Voineagu et al., 2011). These gene expression results are supported by neuroanatomical data showing glial cell activation in ASD postmortem brains (reviewed in Petrelli et al., 2016).

### Distinct pathways deregulated in the BTBR and *En2^−/−^* hippocampus

Phenotype and gene ontology analyses, along with enrichment analyses, also confirmed that marked differences are present between the hippocampal gene expression profiles of BTBR and *En2*^−/−^ adult mice. Mammalian Phenotype ontology analysis showed that the “abnormal innate immunity” and “seizure/abnormal synaptic transmission” pathways are significantly over-represented among hippocampal DEGs in BTBR and *En2*^−/−^ adult mice, respectively (Figure 2). Abnormal immune response has been clearly demonstrated in BTBR mice (see Careaga et al., 2015, and references above). As an example, when pregnant dams of BTBR and C57BL/6J inbred strains were exposed to the viral mimic polyinosinic-polycytidylic acid (polyI:C), severe ASD-like behaviors and persistent dysregulation of adaptive immune system function were only observed in BTBR offspring (Schwartzer et al., 2013).

The gene expression signature of the BTBR hippocampus resulting from the present study is markedly different from that previously published by other authors. We detected a total of 1016 BTBR DEGs, whereas the number of BTBR DEGs detected by Daimon et al. (2015) was significantly lower (301). Only 69 BTBR DEGs were common to the two datasets. In both studies, the hippocampal BTBR transcriptome was compared to that of C57BL/6J inbred mice, and adult mice of comparable age were used (5 months in our study, 4 months in Daimon et al., 2015). However, Daimon et al. (2015) used a different microarray platform (Illumina), microarray analysis software (DIANE 6.0) and pathways analysis software (WebGestalt, http://bioinfo.vanderbilt.edu/), which can justify the difference in the obtained results.

As for the specific gene expression signature of the *En2*^−/−^ hippocampus, KEGG pathway analysis performed in the present study confirmed our previous analysis (Sgadò et al., 2013b). Indeed, a marked excitation/inhibition unbalance is present in the *En2*^−/−^ hippocampus and neocortex, as suggested by the increased seizure susceptibility (Tripathi et al., 2009) and loss of GABAergic interneurons (Sgadò et al., 2013a; Allegra et al., 2014; Provenzano et al., 2014) observed in *En2*^−/−^ mice. These data are also supported by this study (Figure 5), which reveals a significant enrichment of GABAergic/glutamatergic postsynaptic markers among the genes down-regulated in the *En2*^−/−^ hippocampus; genes interacting with fragile X mental retardation protein (FMRP) are also enriched, in keeping with the marked down-regulation of the FMRP pathway detected in the *En2*^−/−^ hippocampus (Provenzano et al., 2015). Finally, dopaminergic markers are also enriched among the genes down-regulated in the *En2*^−/−^ hippocampus, in keeping with the established role of Engrailed proteins in regulating the development and function of dopaminergic neurons (Gherbassi and Simon, 2006). Conversely, microglia markers are significantly enriched among genes down-regulated in the BTBR hippocampus, thus confirming the marked dysregulation of glial cell function in this ASD model. Accordingly, histopathological studies revealed glial cells but not GABAergic neurons alterations in the hippocampus of BTBR mice (Stephenson et al., 2011).

### Weighted correlation network analysis reveals ASD-relevant gene expression modules

Based on the significant similarities detected between the BTBR and *En2*^−/−^ hippocampal DEGs (Figures 2, 3), we considered the gene expression signature of these two ASD mouse models together, and compared it to that of their respective control strains (C57Bl/6J and *En2*^+/+^). We then performed weighted gene co-expression network analysis (WGCNA; Langfelder and Horvath, 2008) to identify correlation patterns among genes across the ASD models vs. control microarray samples, and find gene clusters (modules) whose expression is highly correlated. Genes differentially expressed in ASD mouse models were clustered in 18 modules, and our WGCNA showed that 6 out of these 18 modules were significantly enriched in ASD-related genes. Each of these 6 ASD genes-enriched modules showed a specific enrichment profile in neuronal and glial genes, as well as in genes associated to epilepsy and SCZ. This is in line with WGCNA performed on gene signatures from ASD post-mortem cortical brain tissue, which showed a significant deregulation of glial and neuronal activity-dependent genes in autism (Gupta et al., 2014). It is also important to note that ASD and epilepsy show a high degree of comorbidity (Buckley and Holmes, 2016), and conserved genetic mechanisms have been proposed to underlie ASD and SCZ (see above in this discussion; Stefansson et al., 2014). Thus, WGCNA performed on microarray samples from autistic vs. control mouse strains is able to identify conserved gene expression signatures across different ASD mouse models.

## Conclusions

The present study reveals significant transcriptional similarities and differences between the BTBR and *En2*^−/−^ hippocampus, confirming the idea that transcriptome profiling of specific brain areas from ASD mouse models may contribute to identify novel molecular targets for pharmacological studies.

## Author contributions

GP designed and performed experiments, analyzed data and wrote the paper. ZC and LR performed experiments. KM and TF analyzed data. MS conceived the study and provided funding. YB conceived the study, analyzed data, provided funding, and wrote the paper. ZC and KM equally contributed to this study.

### Conflict of interest statement

The authors declare that the research was conducted in the absence of any commercial or financial relationships that could be construed as a potential conflict of interest.

## Supplementary material

Supplementary Table 1 **Primers used for qRT-PCR analyses**.

Supplementary Table 2 **List of genes differentially expressed in the BTBR hippocampus, as compared to B6 controls**.

Supplementary Table 3 **Raw data of WGCNA analysis on autistic (BTBR and *En2*^−/−^) vs. control (B6 and *En2*^+/+^) differentially expressed genes**.
